# Protein O-GlcNAcylation and the regulation of energy homeostasis: lessons from knock-out mouse models

**DOI:** 10.1186/s12929-022-00851-w

**Published:** 2022-09-04

**Authors:** Tarik Issad, Hasanain Al-Mukh, Abdelouhab Bouaboud, Patrick Pagesy

**Affiliations:** Université Paris Cité, Institut Cochin, CNRS, INSERM, 24 Rue du Faubourg Saint-Jacques, 75014 Paris, France

**Keywords:** O-GlcNAcylation, Metabolic diseases, Glucotoxicity, Inflammation, Oxidative stress, O-GlcNAc transferase, O-GlcNAcase, Knock-out mouse models

## Abstract

O-GlcNAcylation corresponds to the addition of N-Acetylglucosamine (GlcNAc) on serine or threonine residues of cytosolic, nuclear and mitochondrial proteins. This reversible modification is catalysed by a unique couple of enzymes, O-GlcNAc transferase (OGT) and O-GlcNAcase (OGA). OGT uses UDP-GlcNAc produced in the hexosamine biosynthesis pathway, to modify proteins. UDP-GlcNAc is at the cross-roads of several cellular metabolisms, including glucose, amino acids and fatty acids. Therefore, OGT is considered as a metabolic sensor that post-translationally modifies proteins according to nutrient availability. O-GlcNAcylation can modulate protein–protein interactions and regulate protein enzymatic activities, stability or subcellular localization. In addition, it can compete with phosphorylation on the same serine or threonine residues, or regulate positively or negatively the phosphorylation of adjacent residues. As such, O-GlcNAcylation is a major actor in the regulation of cell signaling and has been implicated in numerous physiological and pathological processes. A large body of evidence have indicated that increased O-GlcNAcylation participates in the deleterious effects of glucose (glucotoxicity) in metabolic diseases. However, recent studies using mice models with OGT or OGA knock-out in different tissues have shown that O-GlcNAcylation protects against various cellular stresses, and indicate that both increase and decrease in O-GlcNAcylation have deleterious effects on the regulation of energy homeostasis.

## Background

O-GlcNAcylation was discovered in 1984 by GW. Hart as a new type O-linked glycosylation [1], that was unexpectedly found in the cytosolic and nuclear cell compartments. O-GlcNAcylation has gained considerable interest in biomedical sciences in the last two decades, as it regulates most cellular processes and has been involved in several important human pathologies, including metabolic [2], cardiovascular [3] and neurogenerative diseases [4], as well as cancer [5]. This intriguing reversible post-translational modification regulates a wide range of cellular proteins (more than 4000 proteins identified to date) in a manner reminiscent of phosphorylation [6, 7]. The O-GlcNAc cycling system is apparently simplest than the phosphorylation signalling network, as only two enzymes, OGT and OGA, cloned in 1997 [8, 9] and 2001 [10] respectively, regulate the addition and removal of this sugar from proteins. However, this apparent simplicity brings with itself considerable difficulties for the understanding of its mechanism and specificity of action, both at cellular and whole organism levels [7].

In recent years, numerous studies have tried to explore these issues using OGT and OGA knock-out mouse models. Because O-GlcNAcylation is a post-translational modification tightly dependent on nutritional conditions, and particularly on glucose availability, it plays a determinant role in the regulation of energy metabolism and in metabolic diseases such as diabetes and obesity. In this review, we have analysed the recent literature reporting the consequences of OGT or OGA-KO in mice, but focusing more specifically on those models that have brought important insights into our understanding of the role of O-GlcNAcylation in the regulation of energy homeostasis. We more specifically discuss the apparent contradiction arising from results indicating that whereas increased O-GlcNAcylation level associated with high glucose conditions have deleterious effects, impaired O-GlcNAcylation also markedly alters the regulation of energy metabolism.

### O-GlcNAcylation, a highly dynamic post-translational modification

The regulation of protein activity by post-translational modifications is central to cell signaling mechanisms. Phosphorylation is certainly one of the most studied post-translational modification. However, many other modifications (glycosylation, sumoylation, acetylation, ubiquitinylation, nitrosylation, palmitoylation, ADP-ribosylation, hydroxylation, etc.) are also present on proteins and control their activity, stability and/or subcellular localization. Among these, a particular glycosylation, O-GlcNAcylation, is becoming increasingly important in many fields of biology and biomedical research [6, 7].

Unlike canonical glycosylations, that take place in the endoplasmic reticulum and the Golgi apparatus and mainly concern secreted proteins or extra-cellular domains of membrane proteins, O-GlcNAcylation corresponds to the addition of N-Acetylglucosamine (GlcNAc) on serines and threonines of cytosolic, nuclear and mitochondrial proteins [6, 7]. Like phosphorylation, O-GlcNAcylation is a reversible modification that controls the activity, the stability and the subcellular localization of proteins, as well as their interactions with different cellular partners. Furthermore, O-GlcNAcylation can modulate protein phosphorylation on serines or threonines, either by competing with phosphorylation for the same amino acid or by up-regulating or down-regulating phosphorylation of an adjacent amino acid [11]. However, unlike phosphorylations-dephosphorylations, which are regulated by a myriad of kinases and phosphatases, only two enzymes, OGT (O-GlcNAc transferase) and OGA (O-GlcNAcase) control the addition or removal of the GlcNAc molecule on proteins [12]. OGT and OGA are highly conserved during evolution [8–10]. O-GlcNAcylation is found in most living organisms, with the notable exception of yeast, in which an equivalent has been revealed recently (O-manosylation of cytosolic and nuclear proteins) [13].

### The puzzling question of substrate specificity

**Fig. 1:**
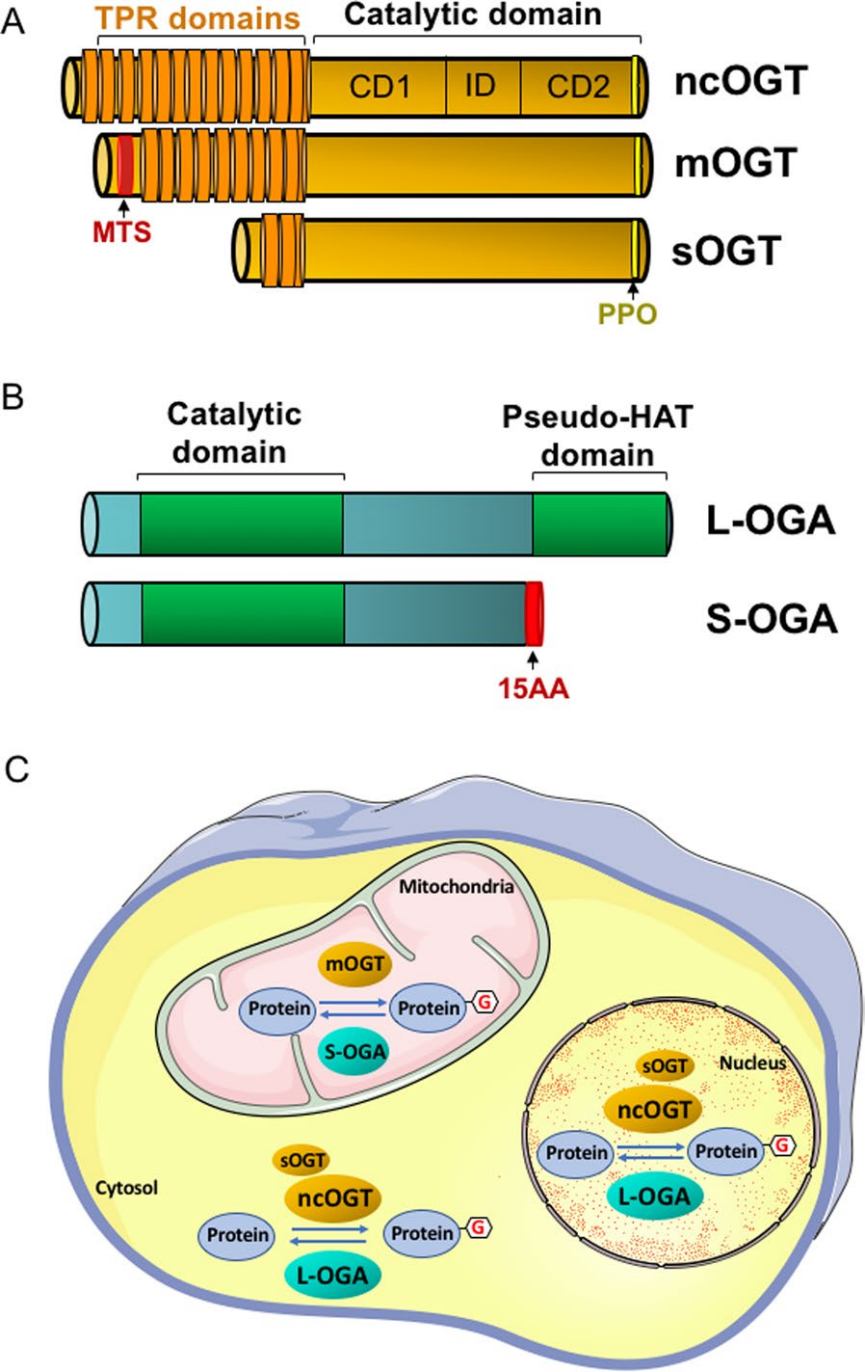
O-GlcNAc-cycling enzymes. **A** Three OGT isoforms, two nucleocyplasmic (ncOGT and sOGT) and one mitochondrial (mOGT), are generated by alternative splicing of the OGT messenger RNA (*MTS* Mitochondria Targeting Sequence). OGT comprises a N-terminal domain containing tetra-tricopeptide repeats (TPR) involved in protein–protein interactions and a C-terminal domain with glycosyltransferase activity. The PPO domain (Phophosphatidylinositol Phosphate binding domain of OGT), located in the C-terminal part, is involved in the recruitment of the OGT to the plasma membrane, allowing its interaction with insulin signaling proteins. *CD1*, *CD2* Catalytic domains 1 and 2. *ID* intervening domain. **B** Two OGA isoforms, long (L-OGA) and short (S-OGA) are generated by alternative splicing of the OGA messenger RNA. L-OGA comprises a hexosaminidase domain in its N-terminal region and a pseudo-histone acetyltransferase domain (HAT) in its C-terminal region. S-OGA does not have the pseudo-HAT domain and seems to be addressed to the mitochondria via a 15 specific amino acids sequence located in the C-terminal region. **C** O-GlcNAcylation is a highly dynamic process regulated by the different OGT and OGA isoforms in different cell compartments

OGT comprises on its N-terminal side a domain containing tetra-tricopeptide repeats (TPR), known to be involved in protein–protein interactions, and in its C-terminal region, the glycosyl-transferase catalytic activity (Fig. 1).

More than 4000 proteins have now been identified as targets for O-GlcNAcylation. A challenging question in the field has been to figure out how a single enzyme, OGT, can accurately regulate such a large panel of substrates in various biological situations.

A certain level of specificity could be achieved by spatial compartmentation of OGT. Thus, 3 different OGT isoforms, arising from mRNA alternative splicing, have been described [14]: a long, nucleocytoplasmic isoform (ncOGT), a short isoform (sOGT) also localized in the cytosol and the nucleus, and an isoform addressed to the mitochondria (mOGT). This spatial compartmentation will restrict the access of the different isoforms of OGT to subsets of specific substrates, for instance intramitochondrial proteins for mOGT [15, 16].

In addition, re-localization of OGT in the vicinity of specific subset of substrates upon hormonal stimulation has been described. Thus, the C-terminal region of OGT comprises a phosphoinsositide binding domain (denominated PPO domain for Phophosphatidylinositol Phosphate binding domain of OGT) which has been involved in the recruitment of OGT at the plasma membrane upon insulin stimulation [17] (see below).

To our knowledge, the potential involvement of PPO in the recruitment of OGT to other cellular membranes has not been investigated. Indeed, increased PIP3 production on endomembranes, including the cytosolic surface of the endoplasmic reticulum and Golgi apparatus, have been observed under different stimuli [18]. Whether the PPO of OGT could also serve for the recruitment of the enzyme to substrates localized on the surface of endomembranes constitutes an important question for future investigations.

As OGT enzymatic activity lacks a strict consensus sequence for substrate recognition, it has been proposed that part of substrate specificity is provided by structural motifs outside of the active site, through the TPR domains located in the N-terminal side of the enzyme. According to this model, different TPR may interact with different substrates, permitting the access of the substrate to the active site of the enzyme. Thus, the deletion of the 3 first TPR of ncOGT had no effect on its activity towards peptide substrates, but totally inhibited its activity towards certain proteins such as caseine kinase II and nucleoporine p62 [19], demonstrating the importance of specific TPR domains for the activity of the enzyme towards specific substrates.

An additional level of specificity may arise from the fact that OGT can also interact with its substrates through adapter molecules, which will recruit substrates to the enzyme in a cell specific and/or context dependent manner. According to this hypothesis, OGT will behave as the catalytic subunit of large transient complexes, where adapter molecules are able to target OGT to its substrates. Thus, in glucose-deprived neuroblastic Neuro-2a cells, p38 MAP kinase targets OGT to the neurofilament protein NF-H, inducing its O-GlcNAcylation and increasing its solubility [20]. In hepatocytes, under fasting conditions (see below), the host cell factor C1 (HCF-1) recruits OGT to O-GlcNAcylate peroxisome proliferator-activated receptor coactivator-1α (PGC-1α) and increase its stability [21], whereas PGC-1α itself has been shown to target OGT to FoxO1 [22].

In addition, post-translational modification of OGT itself can also affect its activity and/or substrate selectivity. Thus, in 3T3L1 adipocytes, tyrosine-phosphorylation of OGT upon insulin stimulation increases its activity, resulting in an increased O-GlcNAcylation of Stat3 [23]. In neuronal NG-10815 cells, potassium chloride-induced depolarization promotes phosphorylation of OGT by the serine-threonine kinase CaMKIV (Calcium/calmodulin-dependant protein kinase IV), resulting in an increase in OGT activity and subsequent stimulation of AP1 transcriptional activity [24]. On the other hand, in liver cells, glucagon-induced calcium signalling promotes OGT phosphorylation by CaMKII, which in turn promotes activation of autophagy in adaptation to starvation [25] (see below).

Two isoforms of OGA [26], long (L-OGA) and short (S-OGA), are also produced by alternative splicing (Fig. 1). L-OGA, more extensively studied than S-OGA, is found mainly in the cytosol and in the nucleus. It comprises the N-Acetyl-glucosaminidase domain in its N-terminal side, and, in its C-terminal side, a pseudo-Histone Acetyl-transferase domain, whose function remains poorly understood.

The less studied S-OGA has only the N-Acetyl glucosaminidase domain and a 15 amino acids C-terminal sequence specific to this isoform. Conflicting results were initially reported regarding the subcellular localization of S-OGA: this isoform was first described as localized in the nucleus of glioblastoma cells [26], then  on the surface of lipid droplets in HeLa cells [27]. However, it has been shown recently that S-OGA is addressed to the mitochondria and is involved in the production of reactive oxygen species (ROS) in this organelle [28]. This strongly supports the notion that O-GlcNAcylation plays an important role in the regulation of oxidative stress [29], at least in part through regulation of mitochondrial ROS homeostasis.

OGT and OGA can both be modified by O-GlcNAcylation, suggesting that cross-regulation can occur between the two enzymes [30]. Moreover, ncOGT and L-OGA have been shown to physically interact within a molecular complex denominated O-GlcNAczyme, and this interaction appears to play an important role in their biological functions [31, 32]. Whether such a complex also exist between mOGT and S-OGA to regulate their biological function in mitochondria remains to be determined.

### The hexosamine biosynthesis pathway

The substrate used by OGT to O-GlcNAcylate proteins is UDP-N-Acetylglucosamine, produced in the hexosamine biosynthetic pathway (HBP).

**Fig. 2:**
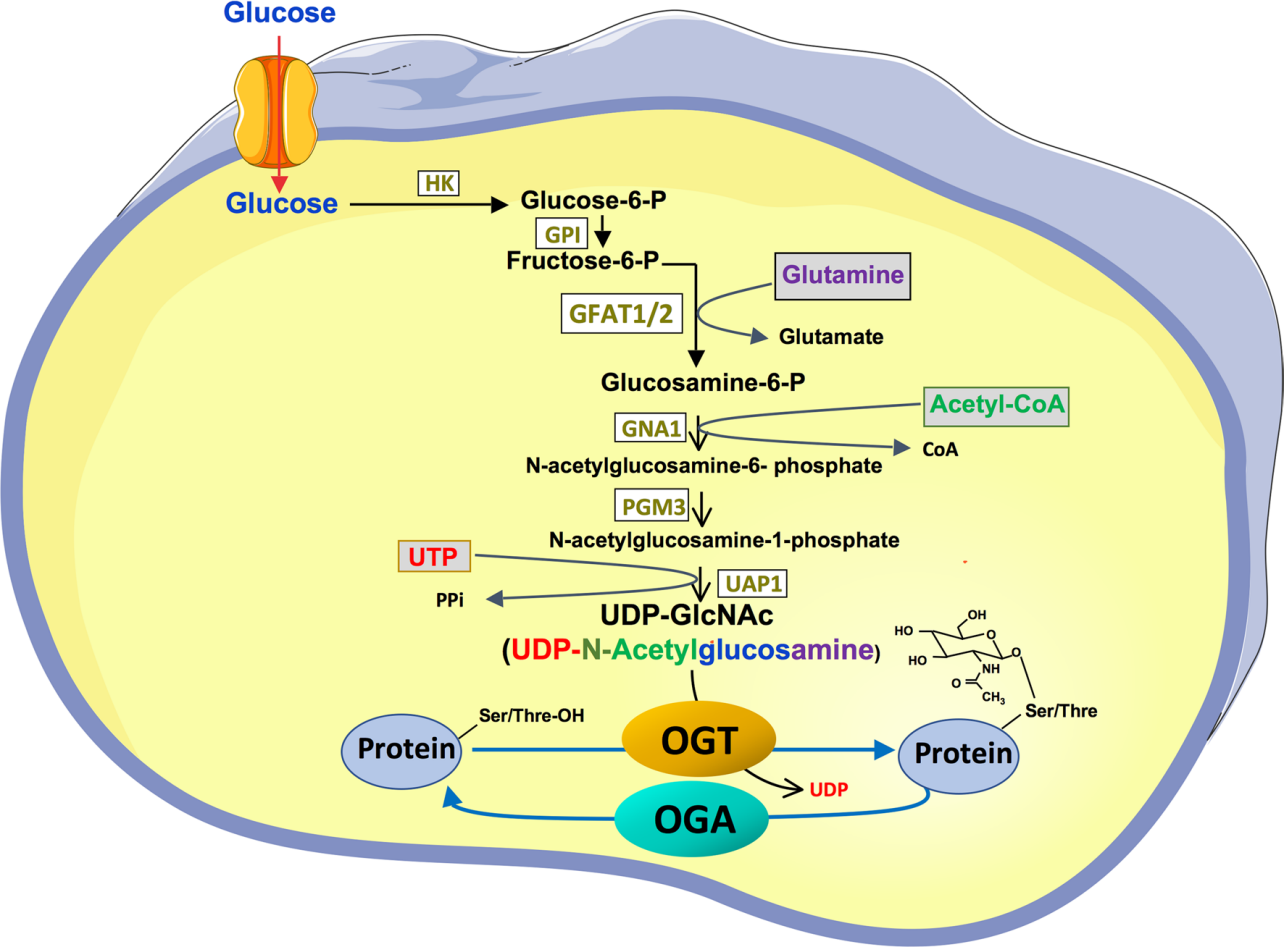
O-GlcNAcylation depends on the energy status of the cell. The hexosamine biosynthesis pathway results in the formation of UDP-GlcNAc, which is used by OGT to O-GlcNAcylate proteins. UDP-GlcNAc is at the crossroads of different cellular metabolisms (glucose, amino acids (glutamine), fatty acids (acetyl-CoA) and nucleotides (UTP)) and therefore reflects the energy status of the cell. *HK* Hexokinase, *GPI* Glucose-6-phosphate isomerase, *GFAT* Glutamine-6-P amidotransferase, *GNA1* Glucosamine 6-P acetyltransferase, *PGM3* Phosphoacetylglucosamine mutase, *UAP1* UDP-N-Acetylhexosamine pyrophosphorylase 1, *OGT* O-GlcNAc transferase, *OGA* O-GlcNAcase

A fraction (2–3%) of the glucose entering the cell is directed to the HBP (Fig. 2). After isomerisation of glucose 6-phosphate into fructose-6-Phosphate in the initial step of glycolysis, fructose-6-phosphate is converted to glucosamine-6-phosphate by the glutamine:fructose-6-phosphate amidotransferase (GFAT), the rate-limiting step of the pathway. After a series of reactions, UDP-N-acetylglucosamine (UDP-GlcNAc) is produced. OGT uses UDP-GlcNAc as a substrate to O-GlcNAcylate nuclear, cytosolic and mitochondrial proteins [12].

GFAT, which catalyses the rate-limiting step of the pathway, is subjected to transcriptional and post-translational regulation. Two GFAT isoforms, GFAT 1 and 2, encoded by different genes (*GFPT1 and GFPT2*), have been described [33, 34]. GFAT1 and GFAT2 have different tissue distribution: GFAT1 mRNA are predominantly expressed in pancreas, placenta and testis, whereas GFAT2 mRNA are expressed in the central nervous system [34]. Differences in the regulation of their enzymatic activities by protein kinase A have been described [35]. However, differential regulation of GFAT1 and GFAT2 expression in different cell types and/or experimental conditions have been poorly explored. As a rate-limiting enzyme, changes in GFAT expression and/or activity are likely to affect UDP-GlcNAc levels [36] and protein O-GlcNAcylation [37].

The hexosamine biosynthetic pathway, which is closely dependent on glucose metabolism, also integrates several other metabolisms: the metabolism of glutamine, which provides the amine function, but also of acetyl-CoA and UTP (Fig. 2). OGT is therefore considered as a metabolic sensor that regulates the activity of proteins according to the energy state of the cell.

It must be noted that changes in intracellular concentrations in UDP-GlcNAc may also participate in the regulation of OGT activity and specificity. Indeed, OGT possesses three different Km for UDP-GlcNAc [38] suggesting that some proteins could be substrates for OGT at low UDP-GlcNAc concentrations, whereas other substrates would be modified only when UDP-GlcNAc concentration in the cell is above a certain threshold. This may constitute an additional mechanism by which OGT could affect the activity of different subsets of proteins, according to metabolic and environmental conditions.

In addition, it should be kept in mind that UDP-GlcNAc is also used for canonical complex glycosylation in the endoplasmic reticulum and Golgi apparatus, and therefore, changes in the activity of the HBP may also affect cellular homeostasis through alteration of complex glycosylation of proteins. Surprisingly enough, cross-talks between canonical and non-canonical glycosylations remain poorly investigated [39].

### O-GlcNAcylation and regulation of glucose homeostasis

Daily nutritional transitions from fasted to fed state imply that important hormonal and metabolic regulations operate to maintain blood glucose concentration within a narrow range. Insulin and glucagon, respectively produced by β- and α-cells of the endocrine pancreas, are two key hormones involved in the regulation glucose homeostasis. In the fasted state, decreased insulin and increased glucagon concentrations in the blood promote liver glucose production, first by glycogenolysis (glycogen hydrolysis), and once glycogen stores are depleted, by gluconeogenesis (synthesis of glucose from lactate, pyruvate and amino acids). This permits endogenous glucose release by the liver under fasting conditions. Upon feeding, increase in blood glucose concentration stimulates insulin and inhibits glucagon secretion by the pancreas. Increased insulin and decreased glucagon inhibit glucose production by the liver, while insulin promotes glucose uptake, metabolism and/or storage in muscle, liver and adipose tissue.

Insulin resistance, defined as a decreased efficiency of insulin action on its target tissues, is a major feature of metabolic diseases, leading to impaired glucose clearance and chronic hyperglycaemia.

It has long been known that chronic hyperglycaemia per se has deleterious effects on various tissues, resulting in further deterioration of insulin sensitivity, impaired insulin secretion, and the development of overt diabetes. Enhanced protein O-GlcNAcylation, resulting from increased glucose availability to feed the HBP pathway, has been suggested as one of the mechanisms underlying the toxic effect of glucose [40]. Indeed, disturbances in protein O-GlcNAcylation have been observed in situations of chronic hyperglycemia, and many studies have shown a role for this modification in the phenomenon of glucotoxicity and in the development of diabetic complications [2]. Consistent with this notion, transgenic animals overexpressing OGT or GFAT in the β cells of the pancreas, the liver, or in muscles and adipose tissue, develop phenotypes reminiscent of obesity or type 2 diabetes (hyperinsulinemia, hyperleptinemia, glucose intolerance, insulin resistance) [41, 42]. Moreover, various studies have indicated that O-GlcNAcylation of different signaling intermediates, such as IRS1 and Akt, negatively regulate insulin signaling and contribute to insulin resistance [17, 43]. An important article has elucidated the mechanism by which OGT regulates proteins involved in early steps of insulin signalling [17]. Upon insulin stimulation, phosphatidyl-inositol 3-phosphate (PIP_3_) production by PI-3 kinase induces the recruitment of OGT, from its nuclear and cytosolic localisation to the plasma membrane. The recruitment of OGT at the plasma membrane through its PPO (Fig. 1A) then favours O-GlcNAcylation of insulin signalling proteins, resulting in attenuation of the signal. Thus, whereas this mechanism may constitute a physiological negative feed-back loop to regulate insulin signaling in the normal situation, under chronic hyperglycemic conditions, an exacerbated O-GlcNAcylation of insulin signaling intermediates would induce a decrease in insulin sensitivity, leading to the establishment of a vicious circle and the aggravation of the hyperglycemia.

In addition to altering early steps of insulin signaling, O-GlcNAcylation activates transcription factors involved in the regulation of carbohydrate metabolism. Excessive production of glucose through liver gluconeogenesis is a major cause of fasting hyperglycemia in diabetes. FoxO1, a major transcription factor involved in the regulation of genes coding for gluconeogenic enzymes, is O-GlcNAcylated and activated under high glucose condition, resulting in increased expression of various gluconeogenic genes [44, 45]. Other transcriptional regulators, including PGC-1α and the CREB regulated transcription coactivator 2 (CRTC2), were also shown to be activated by O-GlcNAcylation, thereby promoting gluconeogenesis in liver cells [22, 46]. O-GlcNAcylation of the transcription factor ChREBP also promotes its transcriptional activity, resulting in increased expression of lipogenic genes and steatosis, thereby contributing to glucolipotixicity in the diabetic liver [47].

Moreover, O-GlcNAcylation appears to be involved in several alterations associated with chronic hyperglycemia, such as cardiovascular and renal complications [2].

Thus, under conditions of hyperglycemia or high nutrient availability, exacerbated protein O-GlcNAcylation can have deleterious effects on several aspects of the regulation of energy metabolism.

However, it should be noted that there is not always a direct relationship between nutrient availability and O-GlcNAcylation level. For instance, in liver cells, HCF-1 recruits OGT to O-GlcNAcylate PGC-1α, thereby facilitating deubiquitination and stabilization of PGC1α. It was shown that O-GlcNAcylation of PGC-1α peaks at 5 mM glucose, whereas it decreased at lower and higher glucose concentrations [21]. Moreover, the same group observed that fasting induces an increase in O-GlcNAcylation and activation of Ulk proteins, promoting liver autophagy in adaptation to starvation [25]. This effect was mediated by a glucagon-induced signaling pathway involving stimulation of calcium/calmodulin-dependent kinase II, increased activity of OGT and Ulk1/2 O-GlcNAcylation, and induction of liver autophagy to maintain energy homeostasis. Another group also described a key role for increased O-GlcNAcylation upon starvation in the liver [48]. Gonzalez-Rellan et al. observed that OGT protein is markedly increased in liver of fasted mice, associated with increased O-GlcNAcylation and stabilization of p53, increased binding of p53 to the *PCK1* promoter and activation of gluconeogenesis. These examples point to the complex role of O-GlcNAc in the regulation of energy metabolism during adaptation to nutritional changes (for a review, [49]).

In agreement with these observations, studies using OGT and OGA knockout mice revealed that both excess and insufficient O-GlcNAcylation can have adverse effects on the control of energy homeostasis.

OGT gene is located on chromosome X, and its invalidation is lethal at a very early stage of embryonic development [50]. Moreover, tamoxifen-inducible global OGT knockdown in adulthood induces a lethal phenotype 4 weeks after induction of the deletion, demonstrating the importance of O-GlcNAcylation for adult mouse survival [51]. To clarify the role of O-GlcNAcylation in vivo, various mouse models with tissue-specific OGT deletion have been developed. OGA is also essential for embryonic development, and its deletion leads to neonatal mortality, indicating the importance of fine-tuned regulation of O-GlcNAc level on proteins [52, 53]. As discussed below, some interesting studies have been performed on hemizygous animals with OGA haplo-insufficiency, but only few models with tissue specific OGA deletion have been developed to date [54, 55].

**Fig. 3:**
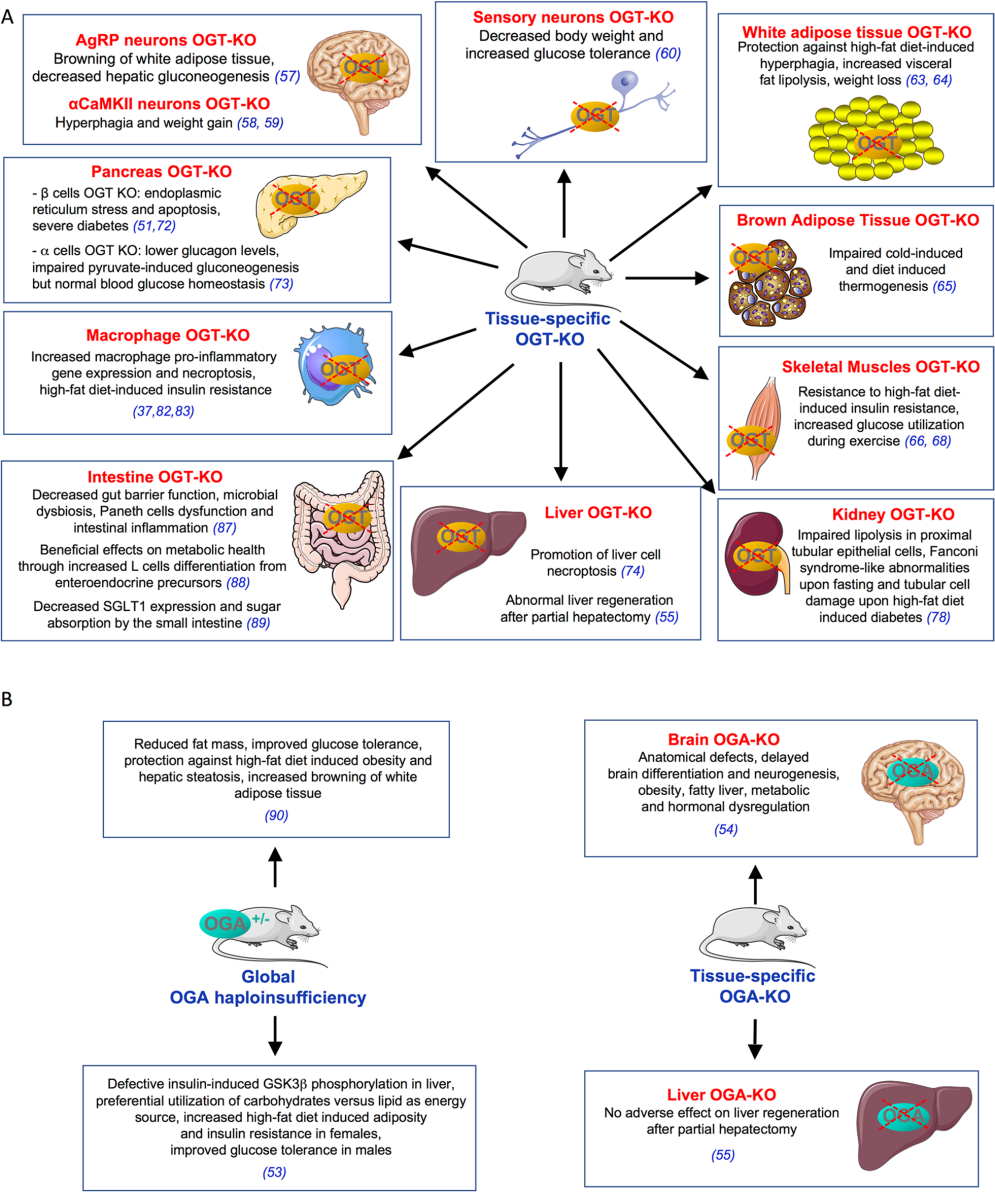
Lessons from OGT and OGA knock-out mice. **A** Tissue-specific OGT-KO mice have brought important insights into the role of O-GlcNAcylation in the regulation of energy homeostasis. **B** Only few tissue-specific OGA-KO mice have been developed to date, and contradictory results have been obtained for the regulation of energy homeostasis in two different models of mice with global OGA haploinsufficiency

OGT and OGA knock-out mice models have brought important insights into the role of O-GlcNAcylation in various physiological and pathological processes. However, in the following paragraphs, we will restrict our discussion to those models that have enlighten the role of O-GlcNAcylation in the regulation of energy metabolism (Fig. 3).

### Lessons from OGT knock-out mice

#### OGT knock-out in the central nervous system

Since OGT is considered as a nutritional sensor, several studies have focused on the potential role of OGT in the central regulation of food intake. AgRP orexigenic neurons are highly activated during the fasting period, in order to stimulate food intake via the inhibition of anorexigenic neurons [56]. Ghrelin is a hormone released upon fasting by the empty stomach that stimulates hunger by activating AgRP neurons in the hypothalamus. Ruan et al. observed that OGT expression as well as protein O-GlcNAcylation levels are increased in AgRP neurons during fasting or upon Ghrelin injection [57], providing an additional example of a situation where protein O-GlcNAcylation and nutrient availability are not correlated. These authors developed mice with selective OGT knockout in these neurons. OGT knockout increases energy expenditure by inhibiting the suppressive activity of these neurons on the browning of white adipose tissue. Moreover, AgRP neurons are known to be involved in the central regulation of hepatic gluconeogenesis, and AgRP neurons-OGT KO mice have decreased hepatic expression of gluconeogenic genes, and a reduction of blood glucose levels during pyruvate and glucose tolerance tests. Consistent with the activated thermogenic program, these mice are protected against high fat diet-induced obesity and insulin resistance. Altogether, this study revealed that in AgRP neurons, OGT has a critical role in suppressing thermogenesis in response to fasting, allowing energy sparing when food intake is reduced, while promoting glucose production through liver gluconeogenesis [57].

Lagerlof et al*.* investigated the effect of tamoxifen-inducible OGT deletion in αCaMKII-expressing neurons [58]. These neurons are mainly located in the paraventricular nucleus (PVN). In this nucleus, food intake activates αCaMKII neurons. Loss of OGT in these neurons completely blocks their activation in response to food intake. Mice knocked out for OGT in these neurons develop hyperphagia leading to increased fat mass and rapid weight gain compared to control mice. This result suggested that in PVN, OGT has a role in the regulation of satiety [58]. Another group [59] independently developed a very similar mice model and confirmed that OGT-KO in αCaMKII-expressing neurons resulted in increased feeding behaviour and obesity within 4 weeks following induction of OGT deletion, associated with higher insulin and leptin levels, insulin resistance and neuronal loss in the hypothalamus. Interestingly, by investigating these mice on a longer period of time, they observed that the insulin resistance reversed 2–3 month after induction of the deletion, and that the mice surprisingly displayed increased insulin sensitivity 9.5 months after neuronal OGT-KO.

Together, these studies [57–59] indicate that suppression of OGT in the brain has different consequences depending on the type of neurons targeted and on neuronal and/or physiological adaptations that may occur on the long-term basis.

#### OGT knock-out in peripheral nervous system

Su and Schwartz [60] invalidated OGT specifically in sensory neurons. These neurons have cell bodies in ganglia along the spine and project sensory axons to multiple organs in the periphery, including the skin, and central axons that enter the spinal cord. These OGT-KO mice were viable but displayed hyposensitivity to thermal and mechanical stimuli, accompanied by decreased epidermal innervation and cell-body loss in the dorsal root ganglia. Interestingly, they exhibit decreased body weight and increased glucose tolerance, but insulin levels were not evaluated, and the metabolic phenotype was not further documented. Further studies will be necessary to determine whether these perturbations can be attributed to altered innervation of pancreas and gut, or to impaired taste and olfactory sensation which would affect feeding behavior.

#### OGT knock-out in white adipose tissue

As mentioned previously, transgenic mouse models have shown that increased flux through the hexosamine biosynthetic pathway induced insulin resistance and hyperleptinemia, but the underlying mechanisms remained poorly characterized. Several lines of evidence have initially indicated that increasing protein O-GlcNAcylation in 3T3L1 adipocytes correlated with inhibition of insulin signaling [17, 43, 61] although the selectivity of the drug used in these studies to increase O-GlcNAcylation (PUGNAc) has been questioned by others, casting doubt on these observations [62].

However, more recent work using adipocyte-specific OGT KO mice has confirmed a crucial role of adipose tissue O-GlcNAcylation in the regulation of adiposity and its involvement in metabolic diseases.

Indeed, Li et *al.* [63] revealed that OGT in adipocytes may link dietary fat sensing to high fat diet-associated hyperphagia. According to this work, OGT would activate, by transcriptional mechanisms, the production of N-arachidonyl ethanolamine (AEA), which acts as an endogenous ligand of the CB1 (cannabinoid 1) orexigenic receptor. The authors revealed that OGT knockout mice in adipose tissue are protected from high fat diet-induced hyperphagia compared to wild-type mice [63].

Furthermore, a recent study by Yang et al. reported that OGT prevents visceral fat lipolysis and promotes central obesity [64]. The authors showed that OGT inhibits lipolysis via O-GlcNAcylation of perilipin PLIN1 (a lipid droplet coating protein), known to regulate lipid metabolism in adipocytes by establishing a physical barrier to triglyceride hydrolysis by adipocyte lipases. Very interestingly, the authors discovered that PLIN1 is O-GlcNAcylated on the same serines that are phosphorylated by PKA during fasting. They observed that OGT knockout results in decreased O-GlcNAcylation and increased PLN1 phosphorylation in visceral adipose tissue, which promotes lipolysis and rapid weight loss, whereas OGT overexpression in mouse visceral adipose tissue inhibits lipolysis and favors diet-induced obesity [64]. In agreement with a role of O-GlcNAcylation in the regulation of adipose mass, the authors showed a correlation between body mass index and OGT/OGA mRNA ratio in human subcutaneous adipose tissue. Furthermore, the OGT/OGA mRNA ratio was significantly higher in subcutaneous and visceral adipose tissue from patients with type 2 diabetes than in adipose tissue from non-diabetic patients, suggesting that elevated expression of OGT in adipose tissue may be a risk factor for obesity and metabolic diseases in humans [64].

#### OGT knock-out in brown adipose tissue

Ohashi et al*.* showed that invalidation of OGT in brown adipose tissue leads to severe cold intolerance [65]. Interestingly, these authors revealed that knockout of OGT induces the degradation of the protein PGC-1α, essential for mitochondrial function (mitochondrial biogenesis, oxidative phosphorylation, oxygen consumption, expression of the uncoupling protein UCP1) [65]. These mice showed hypothermia under fasting conditions, and impaired cold-induced thermogenesis. Moreover, diet-induced thermogenesis by fat feeding was markedly impaired in these mice. Thus, O-GlcNAcylation in brown adipose tissue is crucial to the control of thermoregulation.

#### OGT knock-out in skeletal muscle

In a study on the role of OGT in skeletal muscle, Shi et al*.* first observed that O-GlcNAcylation levels are higher in skeletal muscle biopsies from patients with type 2 diabetes than in biopsies from obese or lean individuals [66]. The authors then studied mice with OGT knock out in skeletal muscle and showed that they displayed a decrease in fat mass, an increase in whole-body energy expenditure, and an increase in insulin sensitivity. These mice were also protected from high-fat diet induced insulin resistance. The authors found that IL15 mRNAs in skeletal muscle and IL15 concentrations in the bloodstream were increased in these OGT-KO mice [66]. The increase in the expression of the IL15 gene was attributed to a decrease in muscle content of an epigenetic repressor (EZH2), which is normally stabilized by O-GlcNAcylation [66]. IL15 is a myokine that promotes resistance to diet-induced obesity and increases insulin sensitivity and muscle oxidative metabolism [67]. Thus, consistent with results obtained with transgenic mouse models overexpressing OGT in muscles [41, 42], these data suggest that OGT in skeletal muscle may promote insulin resistance, at least in part via downregulation of the expression of IL15. However, another mechanism, involving an increase in the expression and activity of AMP kinase in skeletal muscle, associated with enhanced exercise-dependent glucose utilization, has been proposed by Murata et al*.* to explain the increase in insulin sensitivity and resistance to high-fat diet-induced obesity in mice with  muscle OGT KO  [68].

#### OGT knock-out in endocrine pancreatic cells

Several studies have indicated that an increased O-GlcNAcylation in pancreatic β-cells can have deleterious effects on Akt signaling, cell survival and β-pancreatic function [69–71]. However, while chronic increase in O-GlcNAcylation can have a deleterious effect on β-cells, OGT and O-GlcNAcylation appear to be necessary for the proper development of β-pancreatic function. Indeed, mice with OGT-KO in pancreatic β-cells develop severe diabetes due to β-pancreatic insufficiency associated with decreased expression of insulin genes (*ins1* and *ins2*) and transcription factors controlling these genes (Pdx-1 and NeuroD1), decreased PI3K/Akt pathway activity, and β-cell apoptosis induced by endoplasmic reticulum stress [72]. In agreement with these results, Ida et al*.* [51] independently showed that whereas OGT deletion in pancreatic β-cells resulted in higher insulin secretion and hypoglycemia 5–6 weeks after induction of the deletion, the mice became hyperglycemic with insulin depletion and β-cell apoptosis 10 weeks after induction of the deletion.

The role of OGT in pancreatic α-cells has been investigated more recently [73]. These authors observed that mice with targeted OGT KO in α-cells have normal glucose tolerance and insulin sensitivity but significantly lower glucagon levels both in the fed and fasted state. They also exhibit impaired pyruvate stimulated gluconeogenesis in vivo*,* reduced glucagon secretion in vitro, and lower α-cell mass and glucagon content at 6 months of age. Therefore, although OGT appears to be important for α-cell function, OGT deficiency in these cells does not impair blood glucose homeostasis under fed and fasted conditions.

#### OGT knock-out in the liver

One of the major complications of type 2 diabetes is hepatic steatosis (NAFLD for nonalcoholic fatty liver disease). Hepatic steatosis can progress to steatohepatitis (NASH for nonalcoholic steatohepatitis). NASH is characterized by chronic liver inflammation, the appearance of fibrosis, and cell damage, leading to an increased risk of cirrhosis and hepatocellular carcinoma.

As mentioned previously, increased levels of O-GlcNAcylation can have deleterious effects in the regulation of hepatic metabolism, via inhibition of insulin signaling [17] and over activation of gluconeogenic [22, 44–46] and lipogenic pathways [47]. However, OGT also appeared to be necessary for physiological regulation of energy metabolism, particularly in order to promote gluconeogenesis that sustains liver glucose production during fasting [21, 25, 48]. These observations underline the importance of maintaining an optimal level of O-GlcNAcylation for fine-tuned regulation of liver energy homeostasis. Recent data obtained with liver specific OGT knock out mice clearly demonstrated that impaired O-GlcNAcylation in the liver have major deleterious effects. Thus, although mice knocked out for OGT in the liver showed no differences in oxygen consumption, food intake and physical activity, significant tissue alterations (hepatic fibrosis, cirrhosis, inflammation) were observed, related to excessive cell death by necroptosis [74]. Necroptosis is a highly inflammatory form of programmed cell death, independent of caspases, which has been implicated in various liver pathologies including NASH. The exacerbated necroptosis observed in OGT knockout mice in the liver appears to result from the loss of regulation of cellular amounts of RIPK3 (Receptor-interacting serine/threonine-protein kinase 3), one of the key mediators of necroptosis. Indeed, the authors showed that O-GlcNAcylation of RIPK3 by OGT decreases its stability. Thus, OGT deficiency results in increased RIPK3 protein stability, thereby promoting necroptosis [74].

Thus, while over-activation of O-GlcNAcylation in the liver leads to insulin resistance [17] and promotes glucotoxicity [22, 44–47], the absence of OGT can have deleterious effects, via an increase in cell death by apoptosis or necroptosis. These studies revealed an important role for OGT in the protection of the liver against fibrosis and cirrhosis. In addition, in a very recent study, Robarts et al. examined the consequences of OGT deletion in hepatocytes on liver regeneration capacity after partial hepatectomy [55]. In this study, hepatocyte-specific OGT-KO was achieved by injecting OGT-floxed mice with a Cre-expressing Adeno-Associated Virus (AAV8). These authors observed that OGT-KO mice exhibited defects in termination of liver regeneration, with increased liver injury, sustained proliferation, hepatomegaly, hepatic dysplasia and appearance of small nodules 28 days after hepatectomy. Interestingly, this study revealed that failure in termination of liver regeneration was associated with impaired function and stability of HNF4α (Hepatocyte nuclear factor 4α), a master regulator of hepatocyte differentiation, indicating a role for O-GlcNAcylation the regulation of HNF4α activity.

#### OGT knock-out in the kidney

Diabetic nephropathy is a major complication of diabetes, and the crucial role of hyperglycemia in its pathogenesis has been largely demonstrated [75]. Increased HBP and protein O-GlcNAcylation have been involved in diabetic nephropathy, particularly through activation of inflammatory pathways and secretion of extracellular matrix proteins by hypertrophied mesangial cells (smooth muscle-like pericytes that surround the filtration capillaries within the glomerus), resulting in glomerulosclerosis [2]. Renal proximal tubular cells are another cell type affected during diabetic nephropathy development that undergo hypertrophy and interstitial fibrosis, and increased HBP and protein O-GlcNAcylation have also been involved in these alterations[2, 76, 77].

Sugahara et al*.* [78] recently evaluated the consequences of OGT deletion in kidney proximal tubular epithelial cells (PTEC). In these cells, ATP production relies on lipolysis and subsequent fatty acid oxidation in both the fed and fasted states, and disruption of renal lipolysis is involved in diabetic tubulopathy. The authors showed that PTEC-specific OGT-deficient mice had no marked renal phenotype under fed conditions, but display Fanconi syndrome-like anomalies after 48 h of fasting, with high urinary excretion of albumin, glucose, phosphate, uric acid, amino acids and anions, associated with increased apoptosis of PTEC. These alterations were associated with ATP depletion and impaired lipolysis of intracellular lipid droplets in PTEC of OGT-KO mice during fasting, possibly due to decreased level of a cholesterol esterase (CES1), which can also act as a triglyceride lipase. The expression of this enzyme appears to be controlled by O-GlcNAcylation of the transcription factor FXR, and an FXR agonist failed to increase its expression in PTEC isolated from OGT-KO mice. Moreover, the authors showed that OGT depletion in PTEC exacerbates fibronectin accumulation, tubular cell damage and lipid droplet accumulation associated with high-fat diet-induced diabetes. Therefore, whereas hyper-O-GlcNAcylation in diabetic kidney is associated with renal dysfunction, deficiency in O-GlcNAc is also harmful in PTECs, through perturbation of fatty acid metabolism under fasting or diabetic conditions [78].

#### OGT knock-out in the macrophage

Metabolic diseases such as obesity and diabetes are associated with low-grade inflammation, characterized by macrophage recruitment in different tissues, which contribute to alteration in insulin sensitivity and β-cell function [79]. Chronic hyperglycemia appears to play an important role in the establishment and persistence of inflammation, particularly through the promotion of oxidative stress [80]. Several studies indicated that elevated glucose concentrations may also promote pro-inflammatory processes via O-GlcNAcyaltion of different elements in the NFκB pathway (for review, [81]).

However, several recent studies have indicated that inhibition of O-GlcNAcylation in macrophage promotes inflammatory processes. Al-Mukh et al*.* [37] showed that activation of human or mouse macrophages with LPS induces a general increase in protein O-GlcNAcylation. This increase in O-GlcNAcylation, due to a rapid induction of the expression of GFAT2 (the rate-limiting enzyme of the HBP), seems to have a protective effect against inflammatory processes. Indeed, Al-Mukh et al*.* showed that in macrophages invalidated for OGT, the absence of O-GlcNAcylation strongly increased the effect of LPS on the secretion of pro-inflammatory cytokines and on the induction of the enzyme NOS2 (Nitric Oxide Synthase 2) [37]. Li et al. [82] also showed an increase in pro-inflammatory signaling and necroptosis in OGT-KO macrophages. In addition, Yang et al. [83] showed that mice with OGT-KO macrophages exhibit exacerbated inflammation and increased insulin resistance in response to high-fat diet induced obesity. Thus, while some elements of the NFκB signaling pathway are activated by O-GlcNAcylation, O-GlcNAcylation appears to have a protective role against exacerbated inflammation and its deleterious effects on insulin sensitivity. Inflammatory processes are associated with an increase in the production of reactive oxygen species [84], and several studies indicate that O-GlcNAcylation plays an important role in regulating the expression and activity of proteins involved in the oxidative stress response [29]. Interestingly, GFAT2 gene was identified in a genomic screening designed to reveal genes that protect against H_2_O_2_-induced oxidative stress in human HT-22 neuronal cells. This study showed that GFAT2 has a protective effect similar to that of other genes known to be involved in the anti-oxidant response (catalase, glutathione peroxidase-1, peroxiredoxin-1, peroxiredoxin-5, and nuclear factor (erythroid-derived 2)-like 2)[85]. The rapid and robust induction of GFAT2 expression observed upon LPS stimulation in human and mouse macrophages [37] suggests that in macrophages, O-GlcNAcylation may have a protective role in reducing oxidative stress associated with inflammatory processes.

#### OGT knock-out in the intestine

Recent studies have revealed that intestinal dysfunctions, such as enhanced gut permeability and dysbacteriosis-mediated endotoxemia are key players in metabolic diseases associated inflammation [86]. Zhao et al*.* investigated the role of O-GlcNAcylation in the gut [87]. These authors observed a decrease in O-GlcNAcylation and OGT expression levels in colon biopsies from patients with inflammatory bowel disease (Crohn's disease or ulcerative colitis), suggesting a protective role of OGT against inflammation in the gut [87]. The authors also showed that deletion of OGT in mouse intestinal epithelial cells results in decreased gut barrier function, microbial dysbiosis, Paneth cell dysfunction, and intestinal inflammation [87]. Furthermore, the authors showed that deletion of OGT specifically in Paneth cells leads to a dysfunction of these cells, which predisposes mice to colitis chemically induced with DSS [87]. These results revealed the importance of O-GlcNAcylation for the maintenance of intestinal mucosal homeostasis. However, in a follow-up study [88] the same group demonstrated that gut OGT deficiency also have beneficial effects on metabolic health, with decreased body weight and enhanced glucose tolerance. The authors demonstrated that OGT exerts a break on L cells differentiation from enteroendocrine precursors via FoxO1 O-GlcNAcylation. OGT ablation causes L cells hyperplasia, increased secretion of glucagon-like peptide 1 (GLP-1) and disruption of microbial composition that results in improved metabolic control. Thus, the double-edged sword potential of O-GlcNAcylation is clearly revealed by these studies in the gut, with both deleterious [87] and beneficial [88] effects of OGT deletion in the same tissue. A very recent study [89] by Nishimura et al*.* further revealed the complexity of O-GlcNAcylation involvement in the control of intestinal homeostasis. These authors showed that OGT-KO in intestinal epithelial cells resulted in decreased body weight and glycaemia in the fed state, decreased glycaemia excursion during an oral glucose tolerance test, decreased intestinal sugar absorption from the small intestine, associated with reduced expression of the glucose transporter SGLT1. In contrast to the study of Zhao et al*.* [87] which showed marked inflammation in the lower small intestine and in the colon in OGT-KO mice, Nishimura et al*.* found no inflammation in the upper small intestine. These authors suggested that discrepancies between these studies might arise from their focus on different part of the intestine and/or from difference in the gut bacterial flora, which can markedly differ between laboratories [89].

### Lessons from OGA knock-out mice

#### Global OGA haplo-insufficiency

Using gene trap technology, Yang et al. [52] generated global OGA-KO mice by insertion of a trap vector in the first intron of the *OGA* gene. *OGA*^*−/−*^ embryos had stunted growth, reduced size and body weight but did not exhibit gross defects or anatomical abnormalities. However, *OGA*^*−/−*^ newborns died within 1 day of birth, and lung histology revealed markedly reduced alveolar space in these animals. The study of embryonic fibroblasts from these mice indicated that *OGA*^*−/−*^ MEF show proliferative defects and failure in the maintenance of genomic stability. However, *OGA* ^+/-^ were viable and were used by these authors in a follow-up study to evaluate the consequences of *OGA* haplo-insufficiency on energy homeostasis [90]. Unexpectedly, although *OGA* deficiency resulted in hyper-O-GlcNAcylation in muscle, liver and adipose tissue, insulin signaling in these tissues was not affected. Actually, *OGA*^+/-^ had even reduced fat mass and improved glucose tolerance. Moreover, these mice were protected against high-fat diet-induced obesity and hepatic steatosis. Improved energy metabolism was attributed to an increase in energy expenditure through browning of subcutaneous white adipose tissue in these mice, associated with increased PGC-1α and UCP1 protein levels in this tissue, and higher body temperature upon cold exposure [90]. Interestingly, these observations mirror those made by Ohashi et al*.* [65] showing decreased PGC-1α stability, decreased UCP1 expression and reduced thermogenesis in mice with OGT-KO in brown adipose tissue.

Another global OGA-KO mice model was developed by Hanover’s group using a Cre-LoxP-based conditional strategy targeting the promoter and exon 1 of the *OGA* gene [53]. In agreement with Yang et al*.* [52], they observed that *OGA*^*−/−*^ mice showed nearly complete perinatal lethality, although a few animals survived (about 5%). This perinatal lethality was associated with low circulating glucose and low liver glycogen stores. However, in contrast to Yang et al. they observed defective insulin-induced GSK3β phosphorylation in neonatal and adult liver of both *OGA*^+/-^ and *OGA*^*−/−*^ mice. Indirect calorimetry experiments indicated preferential utilization of carbohydrate versus lipid as an energy source in *OGA*^+/-^ compared to *OGA*^+*/*+^ mice. Moreover, a sexual dimorphism was observed upon high-fat feeding, with increased adiposity and insulin resistance in *OGA*^+/-^ female, while *OGA*^+/-^ males did not develop obesity and showed improved glucose tolerance compared to *OGA*^+*/*+^ males [53].

More recently, a knock-in mouse model, in which OGA was replaced with a catalytically-dead mutant enzyme (OGA^D285A^), also showed perinatal lethality and abnormal embryonic growth [91]. This indicates that the phenotype of OGA-KO mice is essentially due to loss of OGA enzymatic glycosidase activity, independently of OGA protein backbone and of its pseudo-histone acetyltransferase domain.

#### OGA knock-out in the brain

The group of Hanover also developed a brain-specific OGA-KO mice model by crossing OGA floxed mice with a mouse line expressing a highly brain-specific *Nes-Cre* transgene [54]. These mice exhibited a pleiotropic phenotype, including anatomical defects, short stature, facial abnormalities, microcephaly, delayed brain differentiation and neurogenesis, reduced cortical and olfactory bulb size and hypotonia. In spite of these perturbations, these mice were generally healthy and female animals were fertile. Total body weight and food intake were not affected. However, these mice exhibited a marked metabolic phenotype including early-onset obesity with increased body fat percentage, fatty liver, and metabolic and hormonal dysregulation (decreased circulating free fatty acids, increased triglycerides, increased insulin, leptin and adiponectin, and decreased IGF1 concentrations). These finding are consistent with the hypothesis that the center of metabolic regulation in the brain, the hypothalamo-pituitary axis, is directly impacted by perturbation of OGA-dependent O-GlcNAc cycling.

#### OGA knock-out in the liver

In the same study on the role of OGT in the regulation of liver regeneration [55], Robarts et al*.* also evaluated the consequences of hepatocyte-specific OGA-KO on liver regeneration in OGA-floxed mice injected with Cre-expressing Adeno-Associated Virus (AAV8). As mentioned previously, the lack of O-GlcNAcylation in OGT-KO liver had profound effects on liver regeneration. In contrast, excess O-GlcNAcylation induced by OGA-KO had no effect on liver regeneration after hepatectomy. As discussed by these authors [55], it is well known that when OGT or OGA expression is modified, a compensatory effect occurs, altering the expression of the reciprocal enzyme [92–95]. However, in liver OGT-KO mice, a compensatory decrease in OGA cannot restore O-GlcNAcylation levels, whereas in liver OGA-KO mice, a compensatory decrease in OGT expression will reduce global O-GlcNAcylation and thereby attenuate the consequences of OGA deletion.

## Conclusion and future directions

Numerous studies, using high-glucose concentrations or pharmacological treatments on cultured cells, as well as transgenic animals overexpressing OGT or GFAT, indicated that chronic increases in O-GlcNAcylation can have deleterious effects and promote phenotypes reminiscent of pathologies such as obesity and type 2 diabetes. However, recent data, obtained with OGT- and OGA-KO mice models, showed that a simplistic scheme in which increased O-GlcNAcylation is considered as deleterious with regard to metabolic diseases does not stand anymore. Consistent with this, knock out of OGT and OGA genes in *C. Elegans* have a similar insulin resistance phenotype, despite having opposite effects on global O-GlcNAcylation levels [96, 97].

O-GlcNAcylation seems essential for an adequate response to various cellular stresses, and maintaining its level within an appropriate range appears necessary for the regulation of cellular homeostasis [7]. This notion is further supported by the tight correlation often observed between OGT and OGA expression levels, permitting to maintain a stable O-GlcNAcylation level within the cell [28, 92–95]. Lessons from OGT and OGA knock out animals indicate that the emerging notion that O-GlcNAc level must be kept within an optimal zone to preserve cellular homeostasis should now be extended to tissue homeostasis and whole-body energy metabolism.

It should also be noted that to date, invalidation of OGT and OGA genes in mouse resulted in loss of expression of all splicing isoforms of these proteins. An important challenge for future research will be to specifically inactivate each isoform, in order to evaluate their function in vivo. Notably, many studies are currently aimed at elucidating the role of protein O-GlcNAcylation in the regulation of mitochondrial functions, which are crucial to the control of energy homeostasis. The recent discovery of a mitochondrial isoform of OGA (S-OGA), involved in the regulation of ROS production by this organelle [28], could open new avenues for the development of molecules with interesting therapeutic potential in the context of metabolic and/or inflammatory diseases. To do so, it will be necessary to fully characterize S-OGA targets in mitochondria, elucidate its mechanism of action in regulating ROS production, develop molecules that will specifically target S-OGA activity or its interaction with its mitochondrial protein partners, and validate these molecules in control and isoform specific knock-out mice.

## Data Availability

All data generated or analysed during this study are included in this published article.
